# A Case Report of *Chlamydia psittaci* Infective Endocarditis Complicated With Pneumonia

**DOI:** 10.1155/crdi/7536462

**Published:** 2025-01-11

**Authors:** Dongmei Zhao, Li Zhang, Qiulin Sun, Jun Cheng

**Affiliations:** Department of Infectious Disease, First Affiliated Hospital of Anhui Medical University, Hefei, Anhui, China

**Keywords:** *Chlamydia psittaci*, infective endocarditis, pneumonia, psittacosis

## Abstract

Psittacosis is a zoonotic disease caused by *Chlamydia psittaci* and is commonly found in birds and poultry. Human infection is uncommon, and most cases are sporadic. Infection of extrapulmonary organs by *Chlamydia psittaci* is extremely rare. A rare case of infective endocarditis complicated by pneumonia caused by *Chlamydia psittaci* was reported, which was diagnosed using metagenomic next-generation sequencing (mNGS). The patient recovered after receiving appropriate anti-infective treatment. Discussion on the pathogenesis, diagnosis, and treatment of this disease based on recent literature reports aimed to improve the prognosis of similar patients and enhance the understanding of clinicians.

## 1. Introduction

*Chlamydia psittaci* is transmitted through close contact with animal species, including infected birds or poultry. Psittacosis typically presents as an acute respiratory symptoms or influenza-like illness. The disease is transmitted through the respiratory tract and presents with nonspecific clinical symptoms, such as fever, cough, expectoration, dyspnea, fatigue, and other clinical symptoms of pneumonia. However, it accounts for less than 5% of community-acquired pneumonia cases [1]. Human extrapulmonary organs are a rare infection caused by *Chlamydia psittaci*. Here, we report a fatal case of infective endocarditis complicated by pneumonia caused by *Chlamydia psittaci* in a woman with a history of rheumatic heart disease and valve replacement surgery. We diagnosed gestational psittacosis during the postmortem study using metagenomic next-generation sequencing (mNGS).

## 2. Case Presentations

The patient, a 67-year-old female farmer, was admitted to the Outpatient Department of Infectious Diseases at the First Affiliated Hospital of Anhui Medical University on September 24, 2022, due to having a fever for one week, cough, and expectoration for two days. One week before admission, the patient developed a fever after experiencing a cold, with a highest body temperature of 39°C, accompanied by chills and joint muscle soreness. However, there was no obvious cough or sputum, and the symptoms persisted despite self-medication with “cold medicine.” On September 20, 2022, the patient experienced a sudden syncope in the morning with no convulsions or incontinences. Following this, the patient was admitted to the Emergency Department of Tongling People's Hospital, whereupon the body temperature was noted to be 38.5°C. Blood routine test indicated that the percentage of the neutrophils was 82.16%, the hemoglobin was 103 g/L, the platelet count was 94 × 10^9^/L, the procalcitonin was 1.73 ng/mL, and the C-reactive protein was 114.25 mg/L. Chest computed tomography (CT) showed a large high-density shadow in the left lung, enlarged heart shadow, and a small amount of left pleural effusion. Head CT showed no obvious abnormality. The patient was given piperacillin sodium and tazobactam 4.5 g intravenously every 8 h for anti-infective treatment. After 2 days of treatment, the patient developed a cough and yellow purulent sputum, and the peak temperature did not decrease. The anti-infective treatment was then adjusted to linezolid 600 mg intravenously every 12 h combined with biapenem 0.3 g intravenously every 12 h and oseltamivir 75 mg orally every 12 h for 2 days. However, the patient's peak temperature still did not decrease significantly, and she experienced chest tightness and abdominal distension after activity.

**Figure 1:**
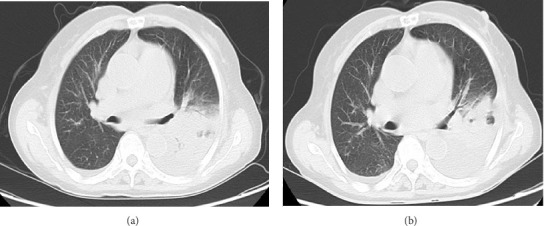
The changes of pulmonary window on CT: (a) and (b) were pulmonary inflammatory lesions at admission and 1 week after treatment. The pulmonary inflammation was more absorbed, accompanied by pleural and pericardial effusion after treatment.

The patient had a history of rheumatic heart disease for more than 20 years, and underwent aortic valve replacement, mitral valve replacement, and tricuspid valve repair in 2010. Long-term oral warfarin 1.25 g everyday, digoxin 0.5 g everyday, and metoprolol 12.5 mg everyday were taken after surgery. Physical examination after admission showed that the temperature was 39.1°C, the breath sounds of both lungs were reduced, and no dry or wet rales were heard. The heartbeat was irregular, and mechanical valve sounds were heard in the valvular areas of the heart. Emergency blood routine showed that the white blood cell count was 3.98 × 10^9^/L, the neutrophil percentage was 88.70%, the hemoglobin was 97 g/L, the platelet count was 92 × 10^9^/L, the procalcitonin was 2.01 ng/mL, the C-reactive protein was 124.62 mg/L, the prothrombin time was 63.4 s, the international normalized ratio was 7.08, and the fibrinogen level was 8.18 g/L. Electrocardiogram showed that the heart rate was 98 beats/min, the atrial fibrillation and T wave changes. Serum antinuclear antibody and ANCA were negative. Serum 1, 3-β-D-glucan test (G test), galactomannan test (GM test), cryptococcal capsular antigen in peripheral blood test, and IgM antibody to respiratory pathogens test (containing *Legionella pneumophila*, *Mycoplasma pneumoniae*, *rickettsia*, *Chlamydia pneumoniae*, adenovirus, respiratory syncytial virus, influenza A virus, influenza B virus, and parainfluenza virus) were negative. Chest CT showed patchy increased density shadows with blurred edges in the left lung, stripe shadows in the left lung and the lower lobe of the right lung, and left pleural effusion (Figure 1).

### 2.1. Diagnostic Procedure

**Figure 2:**
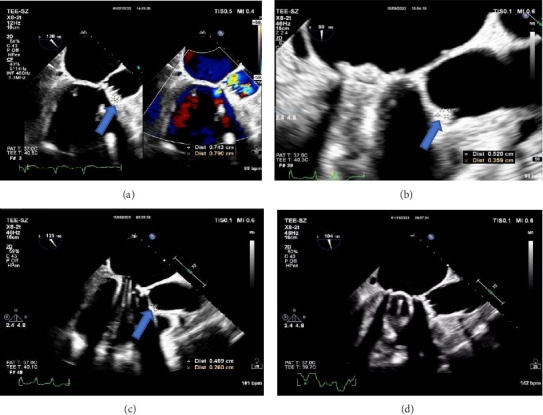
Size change of aortic valve vegetations. (a–c) The size of vegetations was measured at admission, 1 month after treatment, and 2 months after treatment (marked with a blue entity arrow). (d) No vegetations were found in the mechanical aortic valve.

The clinical diagnosis of pulmonary infection was clear, but the etiology was unclear, and the patient's family refused to undergo pulmonary fibrobronchoscopy. Consequently, blood and sputum samples were collected for blood culture, blood mNGS test, and sputum culture. Meropenem 1.0 g was administered every 8 h for 3 days as anti-infective treatment, but the patient's peak temperature did not decrease significantly. Due to significant abnormal results in the coagulation series, warfarin was discontinued, and vitamin K1 30 mg was given intramuscularly everyday along with symptomatic support therapy. The mNGS results received on July 26, 2022, suggested that the pathogen was *Chlamydia psittaci*, detecting one sequence read. Transesophageal echocardiography on September 27 (Figures 2(a) and 2(b)) revealed mechanical dysfunction of the replacement aortic valve and vegetation formation of the replacement aortic valve (0.74 × 0.79 cm light cluster echo attachment of the anterior valve annulus). Supplementary medical history of the patient's family members indicated that several chickens and ducks were raised freely in the open air at home, but no deaths were observed.

### 2.2. Treatment and Outcome

Thereafter, meropenem was discontinued, and the anti-infective regimen was adjusted to minocycline 0.1 g orally every 12 h (double the first dose) combined with levofloxacin 0.6 g intravenously everyday. After 24 h of treatment, the patient's temperature decreased to normal. On October 8, 2022, transesophageal echocardiography was performed again, and the vegetation on the aortic mechanical valve was significantly smaller than before (Figure 2(c)). The patient was discharged on October 10, 2022, and continued to receive minocycline 100 mg orally every 12 h and levofloxacin 0.5 g orally everyday after discharge. The follow-up transesophageal echocardiography on January 11, 2023, revealed the disappearance of cardiac valve vegetation and the absence of new vegetation formation (Figure 2(d)). Antibacterial treatment was stopped, and the patient has been under observation since then.

## 3. Discussion

Psittacosis is an animal-derived infectious disease. Although it has been identified in numerous countries and regions worldwide, often with sporadic outbreaks [2], human infection is uncommon. Following the inhalation of *Chlamydia psittaci* into the lungs, it initially enters the bloodstream, where it proliferates within the mononuclear macrophage system, including the liver, spleen, and lymph nodes, and subsequently disseminates throughout the body via the bloodstream, affecting various organs such as the lungs, liver, spleen, kidneys, and central nervous system. While pulmonary involvement represents the most prevalent clinical symptom, the potential for extrapulmonary infection caused by *Chlamydia psittaci* is often underestimated, particularly in infective endocarditis. This case report demonstrates a remarkably rare occurrence of infective endocarditis caused by *Chlamydia psittaci* with concomitant pulmonary lesions. It delves into the crucial aspects of diagnosis and treatment, offering invaluable insights for standardized clinical diagnosis and treatment approaches (Table 1).

Infective endocarditis caused by *Chlamydia psittaci* has been rarely reported, mainly due to its insidious onset and the challenges associated with detection methods. The laboratory diagnosis of psittacosis primarily relies on culture and serum immunology detection techniques. However, isolating and culturing *Chlamydia psittaci* is demanding, inefficient, and time-consuming. Serum immunology tests exhibit cross-reactivity with other Chlamydiaceae species [3, 4]. In the two cases of infective endocarditis caused by *Chlamydia psittaci* reported in the 1980s, the pathogen was inferred based on the results of serological complement fixation tests, which lacked specificity [4, 5]. In recent years, the emergence of mNGS has offered a novel approach for the clinical diagnosis and treatment, enabling rapid and objective detection of *Chlamydia psittaci* [6–8].

*Chlamydia psittaci* is a gram-negative intracellular parasitic pathogen. The mechanism of cardiac valve infection caused by *Chlamydia psittaci* is still unclear, and it may be attributed to either the direct invasion of the pathogen or an immune-mediated response following infection [9, 10]. Tetracyclines are considered the first-line treatment for *Chlamydia psittaci*, with doxycycline or minocycline commonly used. Macrolide antibiotics can serve as an alternative therapy for pregnant women and children, although the resistance rate of macrolides is high in China [11]. Fluoroquinolones have shown effectiveness in some patients [12]. Adequate intracellular concentrations of antimicrobial agents are crucial for the successful anti-infection treatment of intracellular pathogens. The treatment duration for pulmonary infections caused by *Chlamydia psittaci* typically ranges from 10 to 14 days, but specific treatment plans and durations for infective endocarditis caused by *Chlamydia psittaci* have not been extensively reported. In this case, minocycline combined with levofloxacin was selected for the treatment of infective endocarditis and pneumonia caused by *Chlamydia psittaci*. This combination was chosen due to the favorable tissue permeability of both antibacterial drugs, which is expected to result in a more comprehensive blockage of pathogen protein synthesis, accelerate pathogen clearance, and improve the efficacy of the anti-infection treatment. Based on the disappearance of valve vegetation, the duration of the anti-infection course was approximately 3-4 months. This article aims to provide a detailed diagnosis and treatment approach for rare infective endocarditis caused by *Chlamydia psittaci*, with the intention of offering clinical guidelines for standardized treatment.

In conclusion, *Chlamydia psittaci* infection is not commonly encountered in clinical practice and the clinical symptoms and imaging manifestations often lack specificity. Thus, it is crucial to collect relevant epidemiological data during the process of clinical diagnosis. When considering a respiratory atypical pathogen infection in clinical diagnosis and treatment, mNGS detection of bronchoalveolar lavage fluid or peripheral blood can serve as a supplementary method for the rapid diagnosis of pathogens. Pulmonary infection caused by *Chlamydia psittaci* is the most frequent presentation, with the capability to involve multiple organs simultaneously. However, cardiac valve involvement is rare, emphasizing the need for heightened vigilance in patients with underlying heart valve disease. It is recommended that patients with a history of poultry contact undergo evaluation for infective endocarditis, even in the absence of positive blood cultures.

## Figures and Tables

**Table 1:** The presentation of patient clinical data.

Blood routine	2022.09.24	2022.09.26	2022.09.29	2022.10.08
WBC (10^9^/L)	3.98	3.69	3.63	2.39
NEUT# (10^9^/L)	3.53	2.68	2.53	1.22
LYMPH# (10^9^/L)	0.28	0.55	0.65	0.78
MONO# (10^9^/L)	0.17	0.41	0.34	0.3
RBC (10^12^/L)	3.63	3.19	3.41	3.29
HGB (g/L)	104	90	96	93
PLT (10^12^/L)	92	132	241	176

**Biochemical examination**	**2022.09.24**	**2022.09.26**	**2022.09.29**	**2022.10.08**

ALB (g/L)	32.2		32.5	33.7
GLO (g/L)	29.3		29.1	26.6
TBIL (μmol/L)	11.6		10.5	6.8
ALT (u/L)	52		52	24
AST (u/L)	105		70	32
UREA (mmol/L)	5		8	8
CRE (μmol/L)	77.3		78.5	77.9
UA (μmol/L)	72		81	84
GLU (mmol/L)	9.78		7.62	7.13
LDH (u/L)	681		488	418
CK (u/L)	483		93	89
CKMB (u/L)	11		8	10
K (mmol/L)	3.83	3.42	3.69	4.08
Na (mmol/L)	127.2	136.8	139.6	139.7
CL (mmol/L)	90.7	99.1	100.4	98.9

**Inflammatory indicators**	**2022.09.24**	**2022.09.26**	**2022.09.29**	**2022.10.08**

PCT (ng/mL)	2.01	1.06	0.18	
u-hs CRP (mg/L)	124.62	62.9	21.73	2.11

**Blood cultures or sputum cultures**	**2022.09.24**	**2022.09.25**	**2022.09.26**	**2022.10.04**

	Blood cultures	Blood cultures	Sputum cultures	Sputum cultures
	Negative	Negative	Negative	Negative

		*2022.09.25*		
		NGS (blood)		
		*Chlamydophila psittaci*		

**Chest CT**	**2022.09.24**	**2022.10.02**		

	Diagnostic impression: 1. Inflammation of the left lung; 2. Small nodule in upper lobe of right lung, follow-up; 3. Fibrous foci in both lungs; 4. Bilateral pleural and pericardial effusion	Diagnostic impression: 1. The inflammation of the left lung was slightly absorbed compared with that of the former September 24, 2022; 2. Small nodule in upper lobe of right lung, follow-up; 3. Fibrous foci in both lungs; 4. Bilateral pleural and pericardial effusion; 5. Liver cyst		

**Cardiac ultrasound**	**2022.09.27**	**2022.10.09**	**2022.11.09**	**2023.01.11**

	Diagnosis of transthoracic echocardiography: left atrium and right atrium were enlarged. After mechanical valve replacement of aortic valve and mechanical valve replacement of mitral valve and tricuspid valvuloplasty, mechanical dysfunction of replaced aortic valve suggested the formation of aortic valve vegetations, and further examination by TEE was suggested, triple closure with moderate valve regurgitation was followed up		Diagnosis of transthoracic echocardiography: left atrium and right atrium were enlarged, combined with clinic, mechanical valve replacement of aortic valve, and mitral valve + tricuspid valvuloplasty, tricuspid valve regurgitation, pulmonary hypertension (mild)	Diagnosis of transthoracic echocardiography: left atrial and right atrial enlargement, combined with clinical, mechanical aortic valve and mitral valve replacement + tricuspid valvuloplasty, triple closure with moderate valve regurgitation, pulmonary hypertension (mild)
	Diagnosis of esophageal echocardiography:mechanical dysfunction of aortic valve replacement suggested the formation of vegetations in aortic valve replacement (the anterior annulus of aortic valve showed echo attachment of 0.65 × 0.42 cm). No thrombus was found in atrioventricular and atrial appendages	Diagnosis of esophageal echocardiography: mechanical dysfunction of aortic valve replacement suggested the formation of vegetations in aortic valve replacement (the anterior annulus of aortic valve showed echo attachment of 0.52 × 0.36 cm). No thrombus was found in atrioventricular and atrial appendages	Diagnosis of esophageal echocardiography: after mechanical valve replacement of aortic valve and mitral valve plus tricuspid valvuloplasty, the vegetations of replaced aortic valve were smaller than before (the anterior annulus of aortic valve showed echo attachment of 0.48 × 0.26 cm). No thrombus was found in atrioventricular and auricle	Diagnosis of esophageal echocardiography: combined with clinical, mechanical valve replacement of aortic valve and mitral valve + tricuspid valve plasty, tricuspid valve regurgitation, vegetations in the anterior annulus of the aortic valve disappeared, pulmonary hypertension (mild), and triple closure

**Drug therapy**	**2022.09.24–09.26**	**2022.09.26–10.10**	**Continue treatment after discharge**	

	Meropenem 1.0 g ivgtt Q8H	Levofloxacin 0.6 g ivgtt QD + minocycline 100 mg po Q12H (double the first dose)	Levofloxacin 0.5 g po QD + minocycline 100 mg po Q12H	

## Data Availability

The data that support the findings of this study are available from the corresponding author upon reasonable request.
